# Assessing the Utility of ChatGPT in Simplifying Text Complexity of Patient Educational Materials

**DOI:** 10.7759/cureus.55304

**Published:** 2024-03-01

**Authors:** Rasika Sudharshan, Alena Shen, Shreya Gupta, Sandy Zhang-Nunes

**Affiliations:** 1 Ophthalmology, University of Southern California (USC) Roski Eye Institute, Los Angeles, USA

**Keywords:** spanish-speaking patients, ophthalmology education, health literacy, readability, text simplification, ophthalmology, patient education, chatbots, chatgpt, artificial intelligence

## Abstract

Introduction: AI chatbots are being increasingly used in healthcare settings. There is growing interest in using AI to assist in patient education. Currently, extensive healthcare information is found online but is often too complex to understand. Our objective is to determine if physicians can recommend the free version of ChatGPT version 3.5 (OpenAI, San Francisco, CA, USA) for patients to simplify text from the American Academy of Ophthalmology (AAO) in English and Spanish. This version of ChatGPT was assessed in this study due to its increased accessibility across various patient populations.

Methods: Fifteen articles were chosen from AAO in both languages and simplified with ChatGPT 10 times each. The readability of original and simplified articles was assessed with the Flesch Reading Ease and Gunning Fog Index for English and Fernández Huerta, Gutiérrez, Szigriszt-Pazo, INFLESZ, and Legibilidad-µ for Spanish. Grade levels to assess readability were calculated with Flesch Kincaid Grade Level and Crawford Nivel-de-Grado. Mean, standard deviation, and two-tailed t-tests were performed to assess differences before and after simplification.

Results: Average grade levels before and after simplification were as follows: English 8.43±1.17 to 8.9±2.1 (p=0.41) and Spanish 5.3±0.34 to 4.1±1.1 (p=0.0001). Spanish articles were significantly simplified per Legibilidad-µ (p=0.003). No significant difference was noted for other scales.

Conclusions: The readability of AAO articles in English worsened without significance but significantly improved in Spanish. This may result from simpler syllable structures and a lesser overall vocabulary in Spanish. With increased testing, physicians can recommend ChatGPT for Spanish-speaking patients to improve health literacy.

## Introduction

The Internet is the first source of medical information for almost 75% of individuals in the US [1,2]. While healthcare information is abundantly found online, it is often too complex for patients to understand for many reasons, including lower reading levels, difficulty understanding medical jargon, and limited English proficiency in our population [3-5]. Additionally, with a large population of non-native English speakers in the US, there are increased barriers to health literacy [6-8].

With the advent of chatbots like ChatGPT (OpenAI, San Francisco, CA, USA), users can access information online with greater ease. ChatGPT reportedly gets about 100 million users each week, as per its CEO, Sam Altman [9]. This AI chatbot is advertised as being able to assist with education and clarifying concepts, so it was hypothesized that this capacity could be expanded to medical concepts [10].

Current studies speculate how AI and ChatGPT can be helpful for patient education and have emphasized accessibility for use in populations with limited healthcare access [11,12]. While these studies importantly address improving access to information, our study aims to focus on better comprehension of the abundant materials already available online. As articles on medical advancements continue to get published, there is no tangible change in health literacy, as materials are often far above the average reading level of an adult in the US [3,13,14]. If patients have questions about materials they are referred to, they also do not have an immediate source for reliable answers unless they message their physician or wait until their next visit.

ChatGPT’s free version, version 3.5, has been assessed here as a potential solution to this problem as it is more accessible to a larger patient population. Many patients in the US are Spanish-speaking, and it has been consistently reported that patients who have limited English proficiency are at a disadvantage in not only understanding their conditions but also in communicating with their physicians and ultimately in receiving treatment [7,8,13,15]. Any advances we can make in improving health literacy in this population with this freely available resource can potentially improve health outcomes for these patients.

The American Academy of Ophthalmology (AAO) has already begun to address the gap in limited English proficiency by providing patient education materials readily available in Spanish on their website. Though this is a great start, these materials must be available at reading levels catered to this patient population. In 2021, it was found that 62% of Spanish-speaking patients had less than a high school diploma, and for 44% of these patients, elementary school was their highest level of education [7].

This paper discusses the potential for employing chatbot-based simplification techniques in the realm of healthcare communication. By exploring the advantages, challenges, and potential applications of this technology, we aim to shed light on its transformative role in improving health literacy and overall healthcare outcomes.

## Materials and methods

We assessed the ability of the freely available version of ChatGPT, version 3.5, to improve the readability of educational materials provided by the AAO in both English and Spanish. A preliminary prompt, "What do you do when I ask, “Can you make this text easier to read?”" or "Qué haces cuando yo pienso, “¿Puedes hacer esto más fácil de leer?" was used to confirm that ChatGPT interpreted our subsequent prompt correctly to simplify the text and make the text more comprehensible. This specific phrasing was chosen to model the natural phrasing a patient might use when interacting with ChatGPT while making the objective of improving readability clear to ChatGPT as well.

Fifteen unique educational articles on the same topics in both languages were entered into ChatGPT following the prompt, “Can you make this text easier to read?” or “¿Puedes hacer esto más fácil de leer?" for each of the English and Spanish articles, respectively. Article topics included LASIK, glaucoma, ptosis, age-related macular degeneration, black eye, astigmatism, blue light, color blindness, conjunctivitis, strabismus, dry eye, eye allergies, eyelashes, eyeglasses, and combined cataract surgery and minimally invasive glaucoma surgery. All topics selected for analysis contained a complete article in both languages on the AAO website. Each prompt was repeated 10 times per article, and uniquely generated responses were recorded.

The readability of the original and simplified articles and all ChatGPT outputs were assessed using widely recognized readability scoring formulas. Readability scales are numerical formulas that analyze the characteristics of text, including sentence length, number of words per sentence, syllable count per word, percentage of multisyllabic words, etc. These formulas vary between languages to accommodate differences in language structures. For English text, the Flesch Reading Ease (FRE) Calculator and Gunning Fog Index (GFI) were used. The FRE formula uses mean sentence length (L) and mean syllable count per word (S). FRE scores range from 0 to 100, with larger values indicating higher reading ease [16]. 

FRE Score = 206.835 - (1.015 * L - (84.6 * S)

The GFI formula uses sentence length (L), number of words (N), and percentage of multisyllabic words (M) to calculate readability. GFI scores range from 0 to 20, with larger numbers indicating more difficult readability. GFI values roughly correlate to the number of years of formal education needed to comprehend a given text.

GFI Score = (0.4 * L) + (100 * M / N) 

Spanish readability was assessed using the Fernández Huerta (FH), Gutiérrez, and Szigriszt-Pazo (SP) Calculators and the INFLESZ and Legibilidad-µ (Lµ) scales [17-20]. FH, SP, INFLESZ, and Lµ scores range from 0 to 100, with values closer to 0 being more difficult to read. The FH score is an adaptation of the English FRE and is calculated from the number of syllables (P) and the number of sentences (C).

FH Score = 206.84 - (0.60 * P) - (1.02 * F)

The Gutiérrez score was designed for Spanish texts using the number of characters (L), number of words (P), and number of sentences (F).

Gutiérrez Score = 92.5 - (9.7 * L)/P - 0.35P/F

The SP score uses total syllables (S), total number of words (P), and total number of sentences (C) to calculate the intelligibility of text. The INFLESZ scale is another term for the SP score.

SP Score = 206.835 - (62.3 * S)/P - P/F

The Lµ score uses the total number of words (n), the average number of letters per word (x̄), and the variance (σ²) [17].

µ = [n/(n - 1)](x̄/σ²) * 100

The grade levels of texts were calculated with Flesch Kincaid Grade Level (FKGL) for English articles and Crawford Nivel-de-Grado (CNG) for Spanish articles. Higher values indicate increased reading difficulty. The FKGL formula uses a mean syllable count per word (S) and a mean word count per sentence (W) [20].

FKGL = (0.39 * W) + (11.8 * S) - 15.39

The CNG index calculates reading grade level using the number of sentences per 100 words (OP) and the number of syllables per 100 words.

CNG Score = -0.205OP + 0.049SP - 3.407

Mean, standard deviation, and two-tailed t-tests were performed to assess the difference between the mean readability score of ChatGPT output and that of the original article. All calculations were performed in Excel (Microsoft Corporation, Washington, USA).

## Results

**Figure 1:**
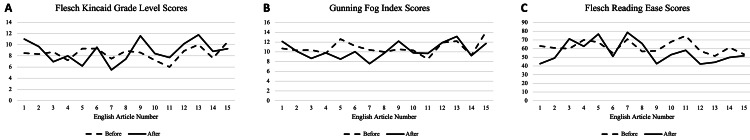
Mean score of individual AAO English articles before and after ChatGPT modification for each readability scoring test Article numbers correspond as follows: (1) LASIK (laser eye surgery), (2) What is glaucoma?, (3) What is ptosis?, (4) What is macular degeneration?, (5) What is a black eye?, (6) What is astigmatism?, (7) Should you be worried about blue light?, (8) What is color blindness?, (9) Conjunctivitis: What is pink eye?, (10) strabismus in children, (11) What is dry eye?, (12) What are eye allergies?, (13) Why are my eyelashes falling out?, (14) Eyeglasses: How to choose glasses for vision correction, and (15) combined cataracts-glaucoma surgery and MIGS AAO: American Academy of Ophthalmology, MIGS: micro-invasive glaucoma surgery

The average grade level of the 15 original English articles was 8.43±1.17 when analyzed with the FKGL formula. Following simplification on ChatGPT, the grade level increased to 8.80±1.88 (p=0.53). The average FRE score for original articles was 61.8±6.96 and decreased to 55.97±12.38 (p=0.12) following simplification. The average GFI score for original articles was 10.81±1.41, while the score for simplified articles was 10.29±1.56 (p=0.34). None of the readability formulas showed statistically significant differences between original and simplified articles in English, and readability scale results per article are shown in Figure 1 and Table 1.

**Table 1:** Mean scores and standard deviation of all tested AAO English articles before and after ChatGPT modification for each readability scoring test. P-values represent the difference between original and simplified means AAO: American Academy of Ophthalmology

	Original Articles		Simplified Articles		
Readability Test	Mean	SD	Mean	SD	P-value
Flesch Kincaid Grade Level	8.4	1.2	8.8	1.9	0.528
Gunning Fog Index	10.8	1.4	10.3	1.6	0.343
Flesch Reading Ease	61.8	7	56	12.4	0.122

**Figure 2:**
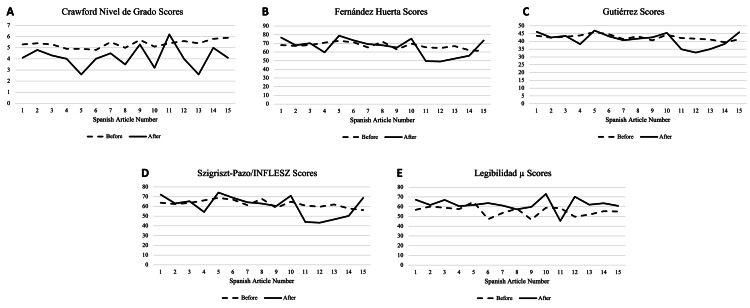
Mean score of individual AAO Spanish articles before and after ChatGPT modification for each readability scoring test Article numbers correspond as follows: (1) LASIK (cirugía ocular con láser, (2) ¿Qué es el glaucoma?, (3) ¿Qué es la Ptosis?, (4) ¿Qué es la degeneración macular relacionada con la edad?, (5) ¿Qué es un ojo morado?, (6) ¿Qué es el astigmatismo?, (7) ¿Debe preocuparnos el uso de la luz azul?, (8) ¿Qué es el daltonismo?, (9) Conjuntivitis: ¿Qué es el ojo rojo?, (10) Estrabismo infantil, (11) ¿Qué es el ojo seco?, (12) ¿Qué son las alergias de los ojos?, (13) ¿Por qué se caen las pestañas?, (14) Gafas: Cómo elegir gafas para la corrección de la visión, and (15) Cirugía combinada de cataratas y glaucoma y MIGS AAO: American Academy of Ophthalmology, MIGS: micro-invasive glaucoma surgery

The average grade level of the 15 original Spanish articles was 5.3±0.34 using the CNG formula. This was reduced to 4.15±1.10 (p=0.0001) after simplification. The average scores for FH before and after simplification were 67.23±3.65 and 65.65±10.99, respectively (p=0.57). Using the Gutiérrez scale, the original articles averaged 42.54±1.68, and they averaged 41.18±4.81 after simplification (p=0.275). Notably, all texts were rated as having normal reading difficulty, with scores around 40. The SP/INFLESZ scales yielded an average score of 62.68±3.76 for the original articles and an average score of 60.61±11.47 for the simplified articles (p=0.476). The average score on the Lµ scale was 55.57±5.02, which increased to 62.34±8.23 after simplification (p=0.003). Readability scale results for articles in Spanish are shown in Figure 2 and Table 2.

**Table 2:** Mean scores and standard deviation of all tested AAO Spanish articles before and after ChatGPT modification for each readability scoring test. P-values represent the difference between original and simplified means. * p<0.005 AAO: American Academy of Ophthalmology

	Original Articles		Simplified Articles		
Readability Test	Mean	SD	Mean	SD	P-value
Crawford Nivel-de-Grado*	5.33	0.34	4.15	1.1	0.0001
Fernández Huerta	67.23	3.65	65.65	10.99	0.57
Gutiérrez	42.54	1.68	41.18	4.81	0.275
Szigriszt-Pazos/INFLESZ	62.68	3.76	60.61	11.47	0.476
Legibilidad-µ*	55.57	5.02	62.34	8.23	0.003

## Discussion

Our results showed that readability was improved in Spanish among this subset of articles but was not improved in English. This is an interesting finding, considering ChatGPT was primarily trained in English. Per ChatGPT itself and OpenAI, this chatbot is advertised as being most proficient in English when compared to the 102 other languages it knows, including Spanish [21]. It is possible that ChatGPT’s inability to significantly improve readability for English articles is due to factors like the English language’s grammar and syntax, the length of words or phrases that cannot be substituted, or ChatGPT’s inability to handle medical terminology. Spanish grammar, for example, does not necessitate the use of a subject at the beginning of a sentence, so removing this extra word from a sentence could alter readability in ways different from English.

Given the relatively recent development of ChatGPT in 2022, there are a limited number of studies investigating ChatGPT’s ability to simplify patient education material. Various articles test across the existing versions of ChatGPT to produce accurate responses to commonly asked patient questions. This was tested in several fields, including glaucoma in ophthalmology, general gastrointestinal disease, and interventional radiology procedures [22-24]. All studies found that ChatGPT can consistently produce accurate patient education information. These studies, paired with our study on the simplification of patient education materials, can prove useful in improving the health literacy of our patients.

Another study published by the American Urological Association compared readability between patient education materials created by urologists and responses from ChatGPT version 3.5. ChatGPT had significantly poorer readability than provider-created articles across all topics that were tested, despite being prompted to provide responses at a sixth-grade reading level (all p<0.001) [25]. It is important to identify opportunities and limitations for chatbot use in patient care settings so we can maximize its impact.

It will be interesting to note if updates to ChatGPT’s free platform will include more training in medical knowledge to further assist in clarifying medical concepts.

To ensure that the goal of improving readability was targeted with the final prompt, ChatGPT was asked how it modified text when given varying phrases like "summarize this text" or "rewrite this text." The wording of the phrase was also chosen carefully based on the perceived likelihood of patients using this phrase. To standardize the results, the final prompt was entered into ChatGPT 10 times, since ChatGPT produced responses with varying reading levels each time. The goal of this study was specifically to assess if ChatGPT can improve readability with one prompt, rather than comparing it across multiple prompts. In the future, more prompts can be used to test ChatGPT's ability to improve readability and perform other functions, such as summarizing text.

While our study has provided valuable perspectives on the ability to use ChatGPT in patient education, it is important to address the following limitations: We used ChatGPT version 3.5 since it is available to patients at no cost and can be recommended for patients with financial barriers. However, with many changes in ChatGPT version 4, it is important to explore the capabilities of the paid version of this tool as well. We also only analyzed a subset of articles from the AAO. These articles from the AAO are specifically designed for patient education, so they may have already started at a lower reading level than other sources of information. In the future, our study should be expanded to test a larger set of articles in different specialties and with other types of medical articles found online to validate consistency. These results should also be compared with those of other freely available chatbots to determine better chatbot recommendations for patients. In general, the average reading levels of patient education materials in English range from 7.2 to 13.4 on the FKGL scale in a 20-year analysis of English patient education materials [3]. The average grade level for the English articles used in this study was 8.4 and 5.33 for the Spanish articles. This should be further investigated, as it is possible that ChatGPT is better at improving readability in articles that start at a lower reading level. As per ChatGPT, when prompted to "make this text easier to read," the chatbot focuses on improving readability by simplifying sentence structure, removing jargon, and clarifying vocabulary. However, the quantitative readability scales used in this study may not adequately assess all the dimensions by which ChatGPT is modifying the text to improve human perception of readability. In the future, patient feedback should be incorporated to support this quantitative analysis and determine if readability has improved. Analyses of ChatGPT's responses should also be performed by ophthalmologists to ensure the accuracy of this material.

## Conclusions

Using existing readability scales, our findings showed that ChatGPT version 3.5 worsened the readability of AAO articles in English without significance and significantly simplified Spanish articles based on two of five scales. While the study showed no improvements in the readability of English-language materials, it highlights a promising avenue to continue exploring the potential of using chatbots to improve healthcare communication with our patients. These findings shed light on the potential for chatbots to be used to improve health literacy among Spanish-speaking patients. With the current results from this study, ChatGPT version 3.5 can be recommended for Spanish-speaking patients to use with AAO ophthalmology articles. However, further exploration should be conducted to analyze whether advancements in chatbots will change our current recommendation or help to expand this recommendation to more patient populations and medical topics.
